# Does Frontal Recess Cell Variation Associate with the Development of Frontal Sinusitis? A Narrative Review

**DOI:** 10.3390/diagnostics14010103

**Published:** 2024-01-03

**Authors:** Tariq Al Habsi, Eiman Al-Ajmi, Mohammed Al Washahi, Maitham Al Lawati, Shihab Al Maawali, Amit Mahajan, Srinivasa Rao Sirasanagandla

**Affiliations:** 1 College of Medicine and Health Sciences, Sultan Qaboos University, Muscat 123, Oman; tariq.alhabsi.a@gmail.com (T.A.H.); maithamlawati.m@gmail.com (M.A.L.); ma3wali2000@gmail.com (S.A.M.); 2 Department of Radiology and Molecular Imaging, College of Medicine and Health Sciences, Sultan Qaboos University, Muscat 123, Oman; ealajmi@squ.edu.om; 3 Department of Surgery, College of Medicine and Health Sciences, Sultan Qaboos University, Muscat 123, Oman; malwashahi@squ.edu.om (M.A.W.); a.mahajan@squ.edu.om (A.M.); 4 Department of Human and Clinical Anatomy, College of Medicine and Health Sciences, Sultan Qaboos University, Muscat 123, Oman

**Keywords:** frontal recess cell, agger nasi, sinusitis, rhinosinusitis, classification

## Abstract

Chronic rhinosinusitis (CRS) can have a significant impact on quality of life. With persistent symptoms and the failure of initial medical treatments, surgical management is indicated. Despite the excellent results of endoscopic sinus surgery for persistent CRS, it is quite a challenging procedure for frontal sinusitis given the complex anatomy and location of the frontal sinus. Frontal recess cells significantly contribute to the complexity of the frontal sinus, and numerous studies have sought to establish their association with sinusitis. This review offers a comprehensive understanding of frontal recess cells, their different classifications, their prevalence among different populations, and their relationship to sinusitis. After an extensive review of the current literature, the International Frontal Sinus Anatomy Classification (IFAC) is the most recent classification method and a preferred practical preoperative assessment tool. Although the agger nasi cell is the most prevalent cell among all reported populations, ethnic variations are still influencing the other cells’ distribution. Studies are inconsistent in reporting a relationship between frontal recess cells and sinusitis, and that is mainly because of the differences in the classification methods used. More research using a standardized classification method is needed to understand the association between frontal recess cells and sinusitis.

## 1. Introduction

Chronic rhinosinusitis (CRS) poses a significant and pressing health issue, demanding our attention and understanding. It is a long-lasting condition that is characterized by inflammation of the nasal passages and the sinuses. This disease generally leads to a variety of symptoms like nasal congestion, headache, difficulty in breathing or nasal block, nasal discharge, decreased sense of smell (anosmia), and postnasal drip. Contrary to acute sinusitis, which usually lasts for a short period, chronic rhinosinusitis persists for 12 weeks or more, due to which patients suffer from reduced quality of life and financial and psychosocial burdens [1,2]. Despite the availability of medical management, surgical options might be indicated for persistent symptoms or failure of initial treatments. Endoscopic sinus surgery (ESS) has shown excellent results in persistent cases [3]. Nevertheless, it is still a challenging technique for frontal sinusitis. In fact, any surgical interventions involving the frontal sinus pose a real challenge because of its unique and complex anatomy [3]. Starting from the location, one factor of concern is that there is a risk of injuring nearby structures like the olfactory apparatus, anterior skull base, anterior ethmoid artery, cribriform plate, and medial orbital wall [4]. The anterior ethmoidal artery, for instance, is an essential anatomical landmark. It is important to be aware of its location at the skull base in the mucous membrane mesentery while performing endoscopic sinus surgeries and to access the frontal sinus. It runs through the roof of the anterior ethmoidal sinus; hence, it is prone to injuries in endoscopic sinus surgeries [5,6]. Another factor of concern is the narrow drainage tract between the orbital and the skull base, which carries the risk of serious complications [3]. The frontal sinus and the space where it drains, the frontal recess, are occupied with diverse cells [7]. Frontal recess cells are groups of air-filled cells found at the anterior ethmoid in the frontal recess. This includes agger nasi, frontal, supraorbital, frontal bulbar, suprasellar, and interfrontal sinus septal cells. They can obstruct the frontal recess outflow, leading to sinusitis [8]. Agger nasi cells, for example, are part of the anterior ethmoidal air cells, and they are the most anterior. These cells are situated anterolaterally and inferiorly to the frontal recess and anteriorly and above the middle turbinate attachment. They are found in 90% of the population and are usually the most common among other frontal recess cells as single cells, but could be found as multiple smaller or larger cells [8]. These variations could be due to attachment of the uncinate process and an enlargement of the ethmoidal bulla, as well as the presence of a large pneumatized frontal beak and crisa galli.

Generally, frontal recess cells show anatomical variations, which can modify the sinus drainage pathway as discussed in the study carried out by Wormald et al., 2016 [7]. There is another study showing the arrangement of these cellular variations along the drainage tract which may increase the chance of obstruction and inflammation [4]. Thus, a comprehensive and clear understanding of this anatomy is required for a better management approach for safe surgery and an excellent outcome [4]. That is why multiple classifications have been proposed for better characterization since 1941 [9]. All these classifications are from a different perspective, which eventually led to a nomenclature discrepancy, and are discussed in detail further ahead in this paper. However, confusion has resulted, given the nature of frontal cells, their complex anatomy, their variations between individuals and populations, and their different categorization methods, making it even more challenging to combine articles from different classifications or even to conclude their association with sinusitis. Therefore, this review attempts to answer whether frontal recess cell variations are associated with frontal sinusitis. In addition, this paper provides an overview of frontal sinus anatomy, the prevalence of frontal recess cells, the different classifications, and factors associated with sinusitis.

### 1.1. Frontal Sinus Anatomy

The frontal sinus is the most superior bilateral paranasal sinus, located just under the forehead, triangular in shape, and located between the outer and inner tables of the frontal bone [10]. The two sinuses are divided by a septum, which usually deviates to either side of the median plane, and they are rarely symmetrical. The aperture of each frontal sinus opens into the anterior part of the middle meatus of the lateral side of the nasal cavity by the ethmoidal infundibulum, either as a frontal recess or directly medial to the hiatus semilunaris [11]. The frontal recess is frequently called the frontal sinus drainage pathway. Frontal sinus drainage through the frontal recess is complex due to the arrangement of air cells surrounding it as well as the attachment of the uncinate process. The frontal recess is bounded posteriorly by the anterior wall of the ethmoidal bulla and anteroinferiorly by the agger nasi [12]. On the lateral side and inferiorly, it is bounded by lamina papyracea and by the terminal recess of the ethmoidal infundibulum, respectively. On the other hand, the frontal recess opens directly into the ethmoidal infundibulum when the uncinate process is attached to the skull base or turned to the medial side [12].

### 1.2. The History of Frontal Recess Cell Classification

**Figure 1:**
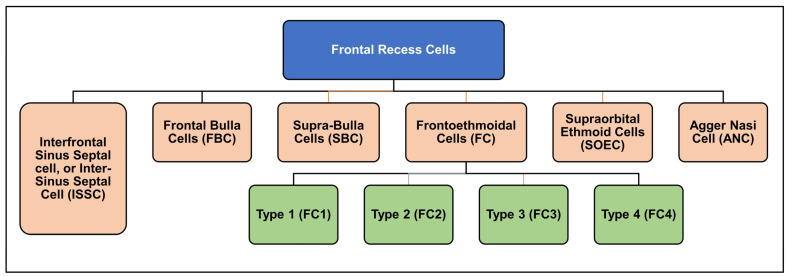
Modified Kuhn classification of frontal recess cells [15].

Before the second half of the 20th century, Van Alyea was the first to group frontal cells into “frontal recess cells” and “invading frontal cells” [9]. In 1994, four different types of frontal cells (frontoethmoidal cells) were identified by Bent et al. [13]. Two years later, the Kuhn classification was introduced [14]. Kuhn classified the cells into six main cells rather than four as compared to Bent et al. [14]. Afterwards, this classification was slightly modified by Wormald and Chan for better clinical differentiation between type 3 (K3) and type 4 cells (K4). The modified Kuhn classification (MKC) was comprehensive and easy to use [15], which significantly facilitated the anatomical understanding of the frontal sinus and nearby structures (Figure 1) [16].

However, there were some limitations reported in terms of names and information redundancy [16,17]. Moreover, the European Position Paper (EPOS) suggested a general way to classify the cells [2,12]. Yet, it lacks cell relationship details which are required in surgery planning and guidance [7]. Recently, two more classifications were developed around the same time to overcome MKC’s limitations. One is the Agger-Bullar Classification (ABC) which is a compartment-based classification rather than one that depends on the cells’ morphology [16]. The other is the International Frontal Sinus Anatomy Classification (IFAC) [7]. The IFAC provided comprehensiveness and more straightforward names using cells’ topographic positions [7,18]. It is the outcome of experts’ consensus internationally. Also, it is a reliable tool after assessing its inter- and intra-rater reliability [17].

The EPOS paper is a re-evaluation of the anatomical terms used and encountered during routine endoscopic sinus surgery [12]. It provides a great summary of the structures within the nasal cavity and the paranasal sinuses. It also compares these terms with the official Terminologica Anatomica [19], providing the available options that can be used in English after a detailed literature review and consensus of experts. It was meant to guide frontal sinus surgeons and to be a reference for general rhinologists. Some of the controversies when describing internal sinonasal anatomy and clarifying areas of confusion were expected to be resolved by this as a reference [12]. Thus, this paper one of the main resources available for such anatomical terminology. Yet, it was not meant to substitute excessive and detailed specialized textbooks in this regard. For the classification of frontal recess cells, EPOS suggested classifying them according to their relation to the frontal recess/inner walls of the frontal sinus, into either anterior or posterior, or medial or lateral locations. Later Wormald et al. [7] proposed a more detailed classification for a better understanding of cell relationships to facilitate surgical planning and interventions.

### 1.3. International Frontal Sinus Anatomy Classification (IFAC)

**Figure 2:**
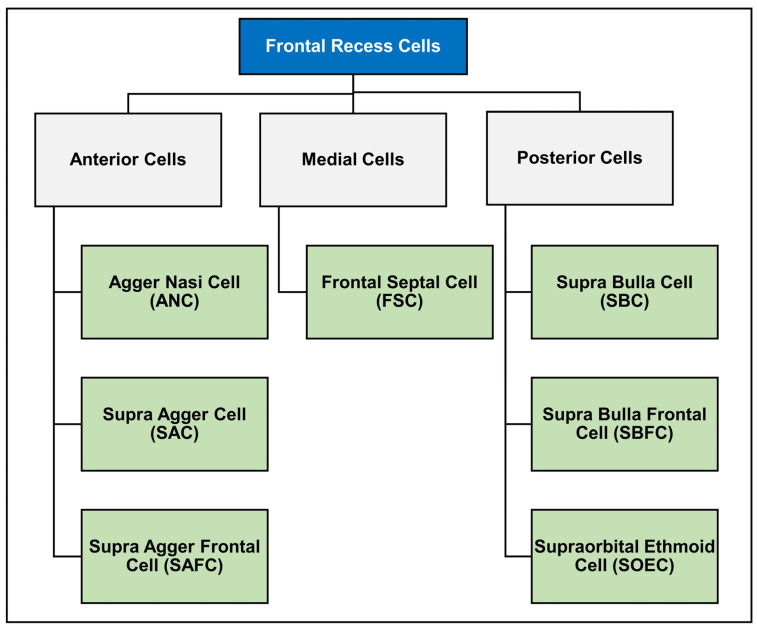
International Frontal Sinus Anatomy Classification (IFAC) [7].

The IFAC addresses the number and position of the cells and their effect on drainage pathways as shown in Figure 2 [7]. Anterior cells shift drainage posteriorly, medially, or posteromedially, these include agger nasi cells (ANCs), supra agger cells (SACs), and supra agger frontal cells (SAFCs). ANCs are the first cell located directly behind the frontal process of the maxilla, near the middle turbinate, whereas SACs lie behind the insertion of the middle turbinate (second part of middle turbinate) to the skull base and are just superior to the ANCs. The middle turbinate is a part of the ethmoid bony complex, located just below the superior turbinate. Its anterior part merges with the agger nasi inferiorly and forms the axilla, while the posterior part attaches with the lamina papyracea and/or medial wall of the maxilla. Superiorly, the middle turbinate attaches with the lateral lamella of the cribriform plate [12].

SAFCs found on the floor of the frontal sinus can be either small or large, depending on the pneumatization into the frontal sinus and the corresponding surgical approach, which can cause obstruction or re-direction of the drainage pathway [18,20]. Supra bulla cell (SBCs), supra bulla frontal cell (SBFCs), and supraorbital ethmoid cells (SOECs) are all posterior frontoethmoidal cells that push the drainage pathway anteriorly. The SBCs lie above the bulla ethmoidalis but do not pneumatize through the frontal ostium, whereas SBFCs are like SBCs with pneumatization through the frontal ostium. Lastly, the SOEC is another posterior cell that is located over the root of the orbit. The final type of cell is the frontal septal cells; they are located medially in the interfrontal sinus septum, thus pushing the drainage pathway laterally or posteriorly [7,18]. The frontal septal cell is referred to as the intersinus septal cell according to EPOS [12].

**Figure 3:**
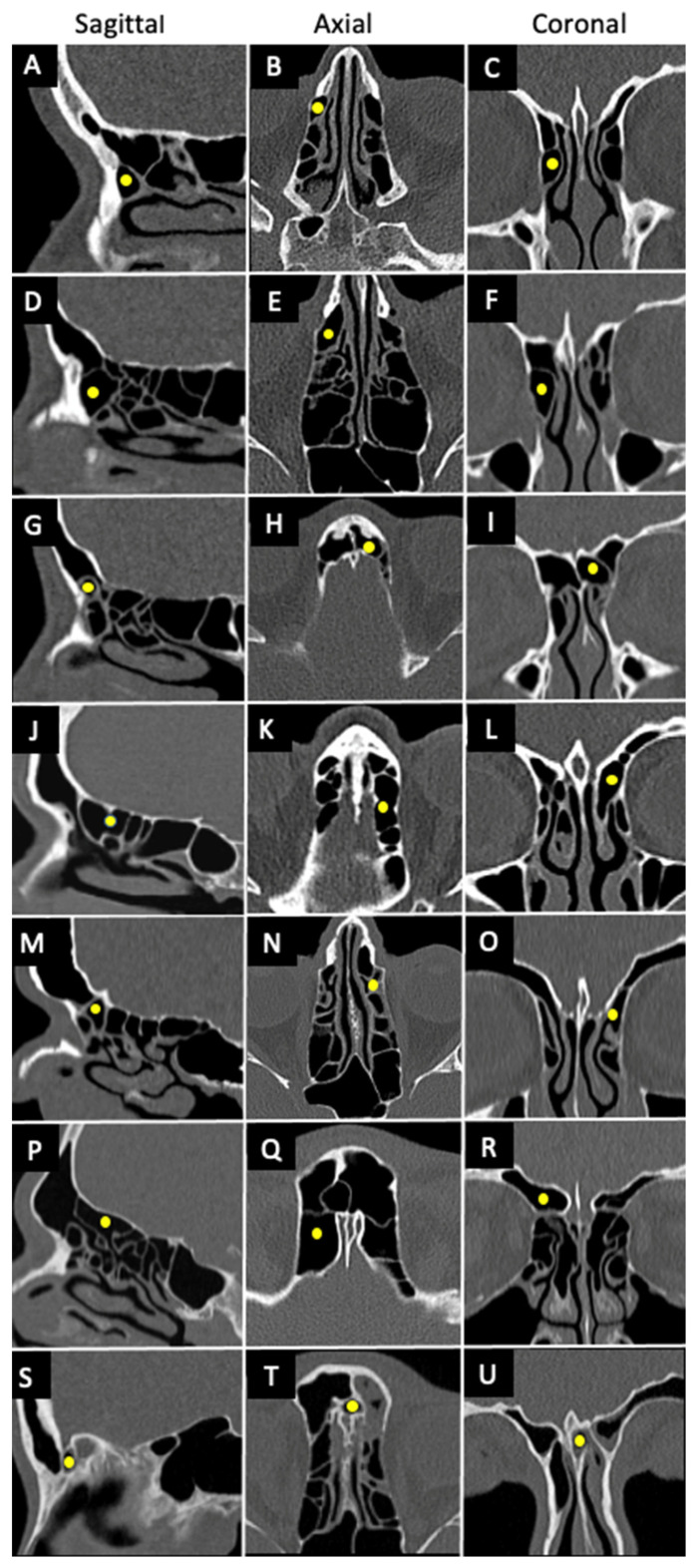
IFAC cell types using computed tomography. Different cell types according to IFAC on CT using sagittal, axial and coronal planes for each cell type: (**A**–**C**) aggar nasi cell, (**D**–**F**) supra aggar cell, (**G**–**I**) supra aggar frontal cell, (**J**–**L**) supra bulla cell, (**M**–**O**) supra bulla frontal cell, (**P**–**R**) supraorbital ethmoid cell, and (**S**–**U**) frontal septal cell.

Figure 3 shows the IFAC cell types in sagittal, coronal, and axial CT scans. 

## 2. Frontal Recess Cells’ Prevalence in Different Populations

Multiple studies investigated frontal cells’ prevalence based on IFAC within different populations [8,18,21,22,23,24,25,26,27] (Table 1). Similarly, other studies investigated their prevalence based on the Kuhn classification [28,29,30,31,32,33] (Table 2). When comparing prevalence results between these populations, ANCs are found to be the highest in all of them. However, the remaining frontal cells’ distribution showed variability [24]. Even when comparing them based on anterior, posterior, and medial groups, there was still no agreement [24]. SBCs’ prevalence was similarly high among Malaysians, Germans, and North Americans, unlike among the Indians and Vietnamese. Almost half or more of the White, Malaysian, German, and Egyptian populations had SACs, while they were lower in the rest. In contrast, SOECs were much lower among Malaysians and Caucasians. Interestingly, Turkish pediatrics showed the highest prevalence in SAFCs and SBFCs, while they were the only group not reporting any frontal septal cells (FSCs). This diversity between different populations might reflect the heterogeneity in different frontal cells [24]. Nevertheless, Fawzi et al. reported that when excluding ANCs, posterior-based groups (SBCs and SBFCs) had a higher prevalence than anterior-based cells (SACs and SAFCs) in previous studies [24], which seems to also be the case with the following studies, except with the White population (Table 1). This supports Fawzi’s argument to classify the cells according to their topographical arrangement rather than individually.

A study [33] compared the frequency of frontal recess cells in Caucasian and Korean subjects using the classification mentioned by Lee et al. [34]. Interestingly, they found the differences between both populations are compatible with their distinctive external facial features, which means that having a more protuberant nasion, glabella, and superior orbital rim was associated with an increased incidence of certain groups of frontal cells, which was the case with Caucasians [33]. They also concluded that these differences in some cells were more likely attributed to ethnic reasons. These were less likely to be related to the difference in the antero-posterior length of the skull base. Howser et al. also supported the link between craniofacial development and the frontal sinus, which might explain the differences between ethnic groups [21]. Furthermore, Johari et al. also compared Malaysian subjects with more than one population using the same classification and they also reported some differences between Southeast Asian and other East Asian populations in some cells [29]. These discrepancies were similarly attributed to their ethnic background. Previous studies show no correlation between the different anatomical variations and the increased incidence of signs of opacification. These variations only alter the surgical approach based on radiological signs and patient symptoms [18].

## 3. Frontal Recess Cells and Their Association with Sinusitis

In regard to the anatomical terminology, authors have used different names for naming the cells surrounding the frontal drainage pathway that include frontal cells [24,28], frontal sinus cells [27], and frontal recess cells [25,29,30,31]. To avoid confusion to the readers, in this review we describe these cells as frontal recess cells throughout the manuscript. The association between frontal recess cell variations and sinusitis has been examined in various studies. Brunner et al. was one of the early studies to examine if agger nasi cells contribute to sinusitis [35]. Although they had a relatively small sample size, they found a significant link between a narrowed nasofrontal duct due to agger nasi cell pneumatization and chronic frontal sinusitis. In addition, Meyer et al. reported that some pneumatization variants significantly affect the presence of frontal cells. In hyperpneumatization, for example, there was a positive association with the appearance of frontal cells and vice versa [36]. It is essential to address the classifications used when discussing what was reported in previous articles. That is because cell identification or labelling might vary according to each model. Thus, to ensure accuracy, we grouped them accordingly. For instance, in 2003, Meyer et al. [36] indicated that individuals with type III and type IV had a significant relationship with frontal mucosal thickening using the Bent et al. classification [13]. Nevertheless, their presence does not always lead to sinusitis [36]. In contrast, three more recent articles following the same classification have yet to find significance [28,37,38]. Several authors have attributed these insignificant findings to sinusitis as a mucosal inflammation rather than an anatomical obstruction [30,31,38].

The following articles used the Kuhn classification [14]. It was challenging to differentiate whether they used the original Kuhn classification [14] or the modified Kuhn classification [15], as the authors did not clarify in most articles. Lien et al. reported that SBCs, FBCs, and SOECs were significantly related to frontal sinusitis, probably due to a narrowed drainage pathway as shortening happens on the anteroposterior parameters of the frontal recess or frontal ostium [31]. They have also reported a significant association with recessus terminalis (RT) due to the absence of a physical barrier along the drainage tract against allergens, irritants, or ascending infections [39]. In the study of Langille et al., although type VI frontal cells were not identified in any subjects, a significance was found with type I, II, and III [40]. It has also been noted that ethnic diversity, seasonality, and the classical presentation of sinusitis are all factors that might explain the variability between different authors [40]. Both Kubota et al. [30] and Johari et al. [29] reported a *p*-value less than 0.05 for only frontal bulla cells (FBCs) with sinusitis. Type III and IV frontal cells were significant in studies by both House et al. and Meyer et al. [36,41]. Interestingly, they also reported a significant *p*-value on the interfrontal sinus septal cell with an odds ratio of 0.51 (0.26, 0.99). This suggests a lesser chance of developing a sinus disease if this cell is present. Lai’s and Hashimoto’s findings indicate no significance when it comes to the presence of these cells [42,43]. On the other hand, they reported a significant association between some opacified areas or opacified frontal cells and sinusitis, respectively. These were the frontal recess and sinus lateralis for the former, and agger nasi, type I frontal cell (FC1), and SBC for the latter.

Since IFAC is a relatively new classification, a few studies only used it to look for its association with sinusitis. In 2019, Sommer et al., using IFAC, attempted to investigate any relationship between the cells and radiological signs of opacification, but the study ended up with no significant findings [18]. However, it is important to keep in mind the way in which frontal cell incidence was reported in this study. It did not differentiate whether a patient had these cells unilaterally or bilaterally, unlike most previous studies, which reported each side separately. One patient can have two cells (one on each side), which might affect the total incidence and the explanation of the results. One year later, Seth et al. failed to draw a significant association. Furthermore, the inconsistency between various studies can be attributed to different ethnicities, classifications, or even a small sample size [25]. Among the IFAC-based articles, only Fawzi et al. found a significant association between developing sinusitis and two types of frontal cells: SOECs and FSCs [24]. It is worth mentioning that both were the least prevalent group of cells in their sample. However, given the position of FSCs, for example, the possibility of frontal sinus blockage can be explained. Thus, their role in sinusitis should be considered, especially during endoscopic sinus surgery. As part of post-surgical management, recurrence is sometimes attributed to an incomplete resection of cells situated within the sinus drainage pathway or a blockage of the drainage pathway [24]. There is an attempt to correlate the IFAC and Kuhn classifications for comparison purposes. Types I and II were assumed to be SACs, and types III and IV were assumed to be SAFCs [26]. Such an assumption must be examined in further studies to reach a clearer conclusion regarding sinusitis and frontal recess cells.

Given all the discrepancies between different classifications and, therefore, different findings, it has been found that having sinusitis can make it even more challenging to identify the cells [44]. On the other hand, the IFAC classification made it easier to assess frontal cells in healthy individuals or those who have a less severe degree of sinusitis [44]. Therefore, concluding an association between frontal cells and sinusitis is limited when severe sinusitis cases are usually excluded due to visualization difficulty [21]. Similarly, Sommer et al. reported the simplicity of using such classification, especially with those with prior anatomical backgrounds [18]. The discrepancies with regard to the association between frontal sinus variations and sinusitis from various studies are presented in Table 3.

### Other Factors Associated with Sinusitis

A number of authors have looked into sinusitis in children [45,46,47,48]. Among this age group, the maxillary sinus was the most involved in sinusitis [45,46,48]. And that could be due to a smaller middle meatus compared to adults [48]. On the other hand, the anterior ethmoid was the most common in adults [48]. April et al. also compared adults and children [45]. They found sinusitis was significantly more frequent in the maxillary, anterior ethmoid, posterior ethmoid, and frontal sinuses in children [45]. However, again among the children group, one of the studies reported no association between their age and the severity of sinusitis [46]. Also, two studies found no relationship between anatomic variations and sinusitis in the pediatric population [46,48]. This might be due to the size of these variations, which do not extend enough to block the sinuses [46]. Others report that this anatomical variation can cause more complications with the sinus, brain/meningeal, or orbit due to simple acute sinusitis [4,49].

In terms of the effect of gender on sinusitis, a study carried out by Lien et al. found no significant relationship [31]. However, more recent evidence revealed a significantly higher sinusitis rate among males with frontal cell type VI [41]. Knowing such differences with gender or ethnicity might decrease the threshold to order dispensable scans [41]. Given all that has been mentioned so far, several authors could not reach a clear conclusion regarding the effect of frontal cells or anatomic variations. Thus, some studies suggested that other causes might be more important than anatomical factors [46,48]. However, overall, there is a consensus that sinusitis is a multifactorial disease. Adding to the anatomical aspect, other factors include mucosal inflammation, sinonasal polyposis, upper respiratory tract infection, allergic rhinitis, adenoid hypertrophy, impaired immunity, gastroesophageal reflux disease, and environmental factors [31,50].

## 4. Surgical Approaches with Anatomical Variations in Frontal Sinus Anatomy

Considering the complexity of the frontal sinus and the overall knowledge of the direct pathway, multiple explanations have been added to address this point. For example, in the presence of a supra agger cell (SAC) with a small bulla cell, the technique is to divert the drainage through the agger nasi window to drain the frontal sinus, while in comparison to the supra- aggar nasi cell with a small bulla cell, the technique is to go with an intact bulla to drain the frontal sinus through an intact bulla with the use of an angled scope and instrument. In contrast, if a large supra agger frontal cell (SAFC) is present, the removal of the bulla and any cells above it is required in order to expose the frontal recess and sinus drainage safely without damaging the orbits, skull base, or anterior ethmoid artery. However, if the nasion is short and low, with a small agger nasi cell, and the drainage is blocked, then entry to the frontal sinus is achieved laterally by drilling the frontal beak in order to face the frontal sinus posterior table using a straight-degree scope and equipment without the need for a curved-degree scope [51]. Further drilling of the frontal beak to the orbital superior medial wall and the medial part of the crisa galli or middle turbinate attachment may be necessary for large supra agger frontal cells and medially supra orbital ethmoid cells, making the procedure Draf 2a/2b or Draf III [7].

## 5. Conclusions

Interventions for frontal sinusitis are considered a surgical challenge given the unique anatomy of this structure. In an attempt to ease this process, different classifications have been developed. Yet, multiple studies reported variations in frontal cells’ prevalence. Others suggested their association with sinusitis. This paper aimed to see if there was any association between frontal recess cells and frontal sinusitis. After carefully reviewing the available literature, there is still inconsistency between the published results. One explanation might be that severe sinusitis cases were excluded due to distorted imaging. Although some significant associations were found with specific groups of cells, the sample size is still insufficient for generalization. Adding to that, some of these associations were related to other anatomical characteristics. Moreover, the ethnic factor plays a crucial role, whether for the prevalence of frontal cells or their association with sinusitis.

Although it is still safe to say that sinusitis is a multifactorial disease, this work enhances our understanding of the frontal recess cells and the factors contributing to sinusitis. The EPOS is a main reference for internal sinonasal anatomy terminology which resolves most controversies and confusion related to the naming of this complex anatomy. Grouping studies according to the classification model used was a beneficial step in analyzing the results. We noticed that the IFAC is a preferred practical classification and preoperative assessment tool. Although it is still a relatively new model compared to Khun, for example, most recent studies rely on it. This is a promising step, as this consensus will standardize the published data. Subsequently, it will help establish a more accurate analysis of future research. Further studies can also investigate the associations of the frontal cells to sinusitis as groups (anterior, posterior, and medial) rather than individually. This approach is more surgically relatable. A natural progression of this work is to assess the association of frontal sinusitis with other anatomical landmarks apart from frontal cells.

## Figures and Tables

**Table 1:** Frontal recess cells’ prevalence among different populations using IFAC method. (ANC: agger nasi cell; SAC: supra agger cell; SAFC: supra agger frontal cell; FSC: frontal septal cell; SBC: supra bulla cell; SBFC: supra bulla frontal cell; SOEC: supraorbital ethmoid cell; CT: computed tomography.)

First Author	Year	Population	Screening Method	Number of Sides	Cell Prevalence (%)						
					ANC	SAC	SAFC	SBC	SBFC	SOEC	FSC
Howser et al. [21]	2023	White	maxillofacial or sinus CT	100	96.0	57.0	26.0	64.0	17.0	12.0	20.0
		Black		100	95.0	40.0	31.0	56.0	17.0	8.0	18.0
		Asian		78	94.9	37.2	23.1	55.1	11.5	21.8	24.4
		Latino		90	97.8	35.6	25.6	54.4	7.8	7.8	16.7
Koksal et al. [22]	2023	Turkish	paranasal sinus CT	160 (Adults)	86.3	35.0	44.4	54.4	46.9	19.4	3.4
				160 (Pediatrics)	93.1	41.9	60.0	76.3	58.5	18.8	0
Nofal & El-Anwar [23]	2022	Egyptian	maxillofacial CT	200	97.0	48.0	11.0	72.0	23.0	42.0	21.0
Fawzi et al. [24]	2022	Malaysian	paranasal sinus CT	400	95.5	50.0	36.0	60.8	53.0	5.5	8.3
Seth et al. [25]	2020	Indian	maxillofacial or sinus CT	180	95.5	33.3	22.2	36.1	21.1	39.4	21.1
Gotlib et al. [8]	2019	Caucasian	paranasal sinus CT	206	86.9	34.0	17.5	77.2	22.8	5.8	27.2
Tran et al. [26]	2019	Vietnamese	sinus CT	208	95.7	16.3	13	46.2	4.3	17.3	10.6
Sommer et al. [18]	2019	German	sinus CT	498	95.2	49	24.9	88.8	26.5	9.2	27.7
Choby et al. [27]	2018	North American	sinus CT	200	96.5	30	20	72	5.5	28.5	30

**Table 2:** Frontal recess cells’ prevalence among different populations using Kuhn method. (ANC: agger nasi cell; FC: frontal cell; FBC: frontal bulla cell; SBC: supra bulla cell; SOEC: supraorbital ethmoid cell; CT: computed tomography.)

First Author	Year	Population	Screening Method	Number of Sides	Cells Prevalence (%)								
					ANC	FC1	FC2	FC3	FC4	SBC	SOEC	FBC	IFSSC
Abraham & Kahinga [28]	2022	Tanzanian	paranasal sinus CT	90	83.3	22.2	35.6	11.1	3.3	N/A	N/A	N/A	N/A
Johari et al. [29]	2018	Malaysian	paranasal sinus CT	312	98.1	28.8	31.1	14.4	0	40.3	16.7	33.0	10.8
Kubota et al. [30]	2015	Japanese	Spiral CT scans	300	88.0	37.0	6.3	4.3	1.3	37.0	6.0	7.0	8.6
Lien et al. [31]	2010	Taiwanese	Spiral CT scans	363	89.0	21.5	10.5	7.7	0	39.1	7.7	6.3	9.6
Han et al. [32]	2008	Chinese	Spiral CT scans	404	94.1	24.4	7.0	8.2	0	36.6	5.4	9.0	12.4
Cho et al. [33]	2006	Korean	sinus CT	114	94.0	22.8	14.0	7.9	0	39.5	2.6	14.0	8.8
		Caucasian		82	86.6	35.4	20.7	8.5	0	11.0	64.6	6.1	7.3

**Table 3:** The association between frontal recess cells and sinusitis from different studies.

Study	Year	Method of Classification Used	Frontal Recess Cell Variation Association with Sinusitis	Sinus Cell Type Associated with Sinusitis
Meyer et al. [36]	2003	Bent	Yes	type III and IV
Delgaudio et al. [38]	2005	Bent	No	N/A
Eweiss & Khalil [37]	2013	Bent	No	N/A
Abraham & Kahinga [28]	2022	Bent	No	N/A
Lien et al. [31]	2010	Kuhn	Yes	SBC, FBC, and SOEC
Langille et al. [40]	2012	Kuhn	Yes	type I, II, and III
Lai et al. [43]	2014	Kuhn	No	N/A
Kubota et al. [30]	2015	Kuhn	Yes	FBC
Hashimoto et al. [42]	2017	Kuhn	No	N/A
House et al. [41]	2017	Kuhn	Yes	type III and IV
Johari et al. [29]	2018	Kuhn	Yes	FBC
Sommer et al. [18]	2019	IFAC	No	N/A
Seth et al. [25]	2020	IFAC	No	N/A
Fawzi et al. [24]	2022	IFAC	Yes	SOEC and FSC

## Data Availability

Not applicable.
